# Medicaid spending on contraceptive coverage and pregnancy-related care

**DOI:** 10.1186/1742-4755-11-20

**Published:** 2014-03-03

**Authors:** François Laliberté, Patrick Lefebvre, Amy Law, Mei Sheng Duh, Jennifer Pocoski, Richard Lynen, Philip Darney

**Affiliations:** 1 Groupe d’analyse, Ltée, 1000 rue de la Gauchetière Ouest Bureau 1200, H3B 4 W5 Montréal, Québec, Canada; 2 Bayer HealthCare Pharmaceuticals Inc., Whippany, NJ, USA; 3 Analysis Group, Inc., Boston, MA, USA; 4 Bixby Center for Global Reproductive Health, University of California, San Francisco, CA, USA

**Keywords:** Medicaid, Spending, Contraceptive, Pregnancy

## Abstract

**Objective:**

Up to 50% of pregnancies are unintended in the United States, and the healthcare costs associated with pregnancy are the most expensive among hospitalized conditions. The current study aims to assess Medicaid spending on various methods of contraception and on pregnancy care including unintended pregnancies.

**Methods:**

We analyzed Medicaid health claims data from 2004 to 2010. Women 14–49 years of age initiating contraceptive methods and pregnant women were included as separate cohorts. Medicaid spending was summarized using mean all-cause and contraceptive healthcare payments per patient per month (PPPM) over a follow-up period of up to 12 months. Medicaid payments were also estimated in 2008 per female member of childbearing age per month (PFCPM) and per member per month (PMPM). Medicaid payments on unintended pregnancies were also evaluated PFCPM and PMPM in 2008.

**Results:**

For short-acting reversible contraception (SARC) users, all-cause payments and contraceptive payments PPPM were respectively $365 and $18.3 for oral contraceptive (OC) users, $308 and $19.9 for transdermal users, $215 and $21.6 for vaginal ring users, and $410 and $8.8 for injectable users. For long-acting reversible contraception (LARC) users (follow-up of 9–10 months), corresponding payments were $194 and $36.8 for IUD users, and $237 and $29.9 for implant users. Pregnancy cohort all-cause mean healthcare payments PPPM were $610. Payments PFCPM and PMPM for contraceptives were $1.44 and $0.54, while corresponding costs of pregnancies were estimated at $39.91 and $14.81, respectively. Payments PFCPM and PMPM for contraceptives represented a small fraction at 6.56% ($1.44/$21.95) and 6.63% ($0.54/$8.15), respectively of the estimated payments for unintended pregnancy.

**Conclusions:**

This study of a large sample of Medicaid beneficiaries demonstrated that, over a follow-up period of 12 months, Medicaid payments for pregnancy were considerably higher than payments for either SARC or LARC users. Healthcare payments for contraceptives represented a small proportion of payments for unintended pregnancy when considering the overall Medicaid population perspective in 2008.

## Introduction

Healthcare costs associated with pregnancy and delivery, and with the care of newborn infants are significant; they represent the two most expensive conditions requiring hospitalization billed to Medicaid in the US [1]. For several decades, low-income women who would not otherwise have been eligible for Medicaid were covered for the care of pregnancy and the post-partum period [2]. As a result, public insurance programs, primarily Medicaid, pay for around half of all births in the United States (US) and for two thirds of all births resulting from unintended pregnancies [3,4], Unintended pregnancies represent approximately 50% of all pregnancies in the US (more than 3 million each year) and are five times more frequent in poor women than affluent women [5]. These unintended pregnancies result in the majority of the 1.2 million abortions per year [6] and are associated with increased risk of detrimental prenatal parental behavior (e.g., substance abuse, short interpregnancy interval) for those carried to term; [7-10] they can also impact women’s health and prospects for education and impose considerable financial burden on families and society [11-13].

The Institute of Medicine and the US Department of Health and Human Services identify contraception as an essential component of preventive health care. Studies on cost of contraceptive coverage relative to pregnancy and maternity care have consistently reported cost savings associated with contraceptive coverage [14-16] Adequate coverage and access to contraception are thus important issues for payers and employers to consider if they support the public health goal of reducing unintended pregnancies and their costs.

Not all types of contraception methods, however, are equally effective at preventing unintended pregnancies [17]. In particular, among contraceptive users, discontinuation and imperfect use of oral contraceptives (OCs) is a leading cause of unintended pregnancy [18,19]. In the recent Contraceptive CHOICE Project, a research study that supports the efficacy of long-acting reversible contraceptive (LARC) methods as a means of reducing unintended pregnancies, participants using oral contraceptive pills, a transdermal patch, or a vaginal ring had an adjusted risk of contraceptive failure that was 20 times as high as the risk among those using LARC [20]. The unadjusted failure rate among participants who used pills, patch, or ring was 4.55 per 100 participant-years, as compared with 0.27 for those who used LARC. The Contraceptive CHOICE Project also reported that when provided with counseling and their choice of contraceptive method at no cost, 67% of eligible women chose an intrauterine device (IUD) or an implant, compared with less than 6% of women in the general population who chose these methods [21].

The Affordable Care Act (ACA) aims to expand Medicaid coverage to millions of low-income Americans. The ACA Medicaid expansion may be costly for some states [22], but it could also lead to potential cost-offsets since Medicaid includes contraceptive coverage which should result in fewer unintended pregnancies. This retrospective study of health insurance claims used multi-state Medicaid data to evaluate all-cause healthcare payments for women using different types of contraception and for pregnant women. The relative spending on contraceptive methods was also compared. Moreover, payments on contraception and on pregnancy care among all Medicaid beneficiaries were assessed, as well as the estimated payments on unintended pregnancies. We hypothesized that the costs of contraceptive coverage to a health plan are considerably lower than payments for unintended pregnancy care and that LARC methods provide maximum cost savings.

## Methods

### Data source

We analyzed the combined Medicaid health claims data from 2004 to 2010 for five states (Florida, Iowa, Kansas, Missouri, and New Jersey) for whom we had access to their Medicaid data. The Medicaid database used contains complete medical and pharmaceutical claims for over 11 million Medicaid beneficiaries including Medicare/Medicaid dual eligible beneficiaries. As showed in Table 1, the demographic characteristics (e.g., age groups, race) of women Medicaid beneficiaries from the combined studied states were representatives of the demographic of all women Medicaid beneficiaries in the US as evaluated in 2008. The database includes information on enrollee eligibility, physician visits, hospitalizations, long term care services, and prescription drugs. Of note, in addition to standard demographic variables such as age and gender, the database includes variables such as aid category (blind/disabled, Medicare eligible) and race. The medical claims also contain diagnosis and procedure information, and the prescription drug claims contain information on the name, dosage, formulation, and days of supply of the medication as well as the amount of the Medicaid payment for each claim.

**Table 1:** Descriptive statistics on women medicaid beneficiaries in the studied States (NJ, FL, MO, KS, and IA) compared to medicaid beneficiaries in the US in 2008

**Beneficiaries characteristics**	**Medicaid beneficiaries from the combined studied states (NJ, FL, MO, KS, and IA) in 2008**	**Medicaid beneficiaries in the US in 2008**^**1**^
Women beneficiaries (%)	58.4%	59.0%
Age of women beneficiaries (%)		
< 15 years	37.4%	34.3%
15-18 years	7.5%	7.8%
19-44 years	30.5%	35.8%
45 or more years	24.6%	22.2%
Race of women beneficiaries (%)		
White	45.1%	41.3%
Black	25.8%	22.3%
Hispanic/Latino	17.9%	21.4%
Other	11.2%	15.0%

Note:

1. Obtained from the Medicaid Statistical Information System (MSIS) State Summary Datamart. Available at http://www.cms.gov/Research-Statistics-Data-and-Systems/Computer-Data-and-Systems/MedicaidDataSourcesGenInfo/MSIS-Mart-Home.html. Accessed January 10, 2014.

Medicaid database are de-identified and fully compliant with all Health Insurance Portability and Accountability Act of 1996 privacy and security requirements to protect participant anonymity and confidentiality. Institutional review board (IRB) approval and informed consent were not required for this study.

### Study design

The first set of analyses (Retrospective Cohort Design) focused on payments made on behalf of Medicaid-covered women of reproductive age who were either initiating contraceptives – whether short-acting reversible (SARC: OC, transdermal, vaginal, or injectable) or LARC (intrauterine device [IUD] or implant) – or became pregnant during the study period. In addition the analyses set out to compare spending on different types of contraceptives compared to oral contraceptives. A second set of analyses (Actuarial Analysis) examined these payments in relation to all Medicaid enrollees included in the study regardless of gender and utilization of services. Of note, the SARC and the LARC contraceptive methods were studied individually (OC, IUD, etc.).

#### Retrospective cohort design

A retrospective cohort design was used to evaluate contraception and pregnancy healthcare payments for Medicaid-covered women of reproductive age during up to 12-months of follow-up. An observation period of only up to 12 months was chosen to take into account the high discontinuation rate among SARC users [18,19] and the fact that some women who initiated contraceptive methods discontinued later because they wanted to get pregnant. A follow-up of 12 months was also appropriate to evaluate pregnancy healthcare payments.

Women 14–49 years of age enrolled for ≥6 months prior to initiating SARC or LARC contraceptive methods and pregnant women were included as separate cohorts. Each woman was followed from the first contraceptive claim (contraceptive cohort) or from the first pregnancy or pregnancy-related diagnosis (pregnancy cohort) claim, until the earliest of 12 months of follow-up, health plan disenrollment, or end of data availability. Of note, since we studied real-world contraceptive use and pregnancy occurrences, the contraceptive-users cohorts could include women who became pregnant (either because they wanted to become pregnant or because they had an unintended pregnancy). It is also possible that a patient initiated a contraceptive and then became pregnant, in which case that patient would be in both a contraceptive cohort and in the pregnancy cohort. Similarly, by studying real-world usage, women were allowed to switch contraceptive methods during the up to 12 months of follow-up.

Medicaid spending for each cohort of contraceptive users up to 12 months after the index date was summarized using mean all-cause healthcare payments and contraceptive payments. All-cause, pregnancy-related, and pregnancy-related complication payments were calculated for the pregnancy cohort. Pregnancy-related payments were identified as hospitalizations and outpatient claims with a primary or a secondary diagnosis for normal pregnancy (ICD-9-CM: V22), supervision of high-risk pregnancy (ICD-9-CM: V23), outcome of delivery (ICD-9-CM: V27), normal delivery (ICD-9-CM: 650.x), and complications of pregnancy, childbirth, and the puerperium (ICD-9-CM: 630.×-676.×). Pregnancy-related complications payments, a subset of pregnancy related payments, included complications of pregnancy, childbirth, and the puerperium. Neonatal care payments were not included in pregnancy-related payments since the focus of this study was on the costs of the pregnancy. Adding neonatal care in pregnancy-related payments would considerably increase pregnancy-related costs, which makes our estimated cost of pregnancy conservative.

#### Actuarial analysis

Since women can be expected to be on contraception for many years, while women who become pregnant become so at a fairly low rate during the same time frame with a limited number of pregnancies being carried to term, an actuarial analysis was also conducted that has the advantage of evaluating contraception and pregnancy healthcare Medicaid payments for both new and ongoing contraceptive users, as well as newly pregnant or ongoing pregnant women.

The study population for this analysis included all Medicaid members (i.e., all females regardless of contraceptive use and pregnancy status and all males) with at least one month of Medicaid coverage in 2008. No other inclusion or exclusion criteria were applied. Each member was followed from the first day of eligibility in 2008 until the earliest of December 31, 2008, or health plan disenrollment. For example, if a member was enrolled in Medicaid before 2008 and was eligible until the end of March 2008, this patient was studied from January 1, 2008, through March 31, 2008. A member enrolled July 1, 2008 and still eligible at the end of 2008 was studied from July 1, 2008, through December 30, 2008. The subset of females of childbearing age (14–49 years) was also studied.

### Statistical analysis

For the 6-month period preceding the index date, patient baseline characteristics were described with means and standard deviations for continuous variables and with frequencies and percentages for categorical variables.

For the retrospective cohort analysis, Medicaid spending for each cohort was summarized using mean healthcare payments per patient per month (PPPM). The PPPM is the aggregated payment divided by aggregated months of the follow-up period, with both values summed across all patients, an approach commonly used in non-experimental study settings to account for different lengths of observation periods among study patients. The PPPM cost is a useful measure to evaluate the costs among a group of patients since it evaluates the mean cost per month among these individuals.

To evaluate all-cause costs and contraceptive costs of OC users compared to other contraceptive users, ordinary least square regressions were used in both univariate and multivariate analyses to evaluate unadjusted and adjusted payment differences. Confidence intervals (95% CI) were calculated using a nonparametric bootstrap to account for the non-normal distribution of data. Covariates included for adjustments in the multivariate analysis were age, region, race, year of index date, Charlson comorbidity index, other comorbidities (cardiovascular diseases, hypertension, diabetes, pelvic inflammatory disease, alcohol, smoking), parous status, and baseline healthcare payments. The Charlson comorbidity index is a measure of the sickness of patients that is commonly used in claims analyses. Each of the 17 conditions (e.g., congestive heart failure, renal disease, cancer) in the Charlson comorbidity index is assigned a score and the total score of each patient was evaluated and calculated during the 6-month baseline period [23]. Baseline healthcare payments represented the total Medicaid payments for patients during the 6-month baseline period prior to the index date.

For the actuarial analysis, Medicaid payments for all contraceptives, OC, IUD, and pregnancy care in 2008 per female member of childbearing age per month (PFCPM) and per member per month (PMPM) were evaluated. These metrics are different from the PPPM method described above since they evaluated the costs not just among women in the contraceptive or the pregnancy cohorts, but in a larger group of women regardless of their contraceptive usage in the case of the PFCPM analysis and in an even larger group that included all Medicaid members (female or male) for the PMPM analysis. PFCPM payments were calculated as the total payments for women aged 14 to 49 divided by patient-months of observation. Similarly, PMPM payments were calculated as PFCPM payments but divided by all Medicaid beneficiaries, thus giving a perspective of the same payments, but on all Medicaid beneficiaries instead of just female members of childbearing age. An individual could thus be enrolled for only part of the year. Retrospective actuarial analyses using similar patient-year calculations have also been conducted in previous studies [24,25].

In addition, Medicaid payments for unintended pregnancy were also estimated based on rates of unintended pregnancies reported in the literature for Florida (59%), Iowa (44%), Kansas (48%), New Jersey (55%), and Missouri (53%) [26]. By applying these proportions to corresponding pregnant women included in the current study, we obtained a combined unintended pregnancy rate of 55%.

All payments were inflation-adjusted to 2011 U.S. dollars based on the medical care component of the Consumer Price Index.

## Results

### Patient characteristics

**Figure 1:**
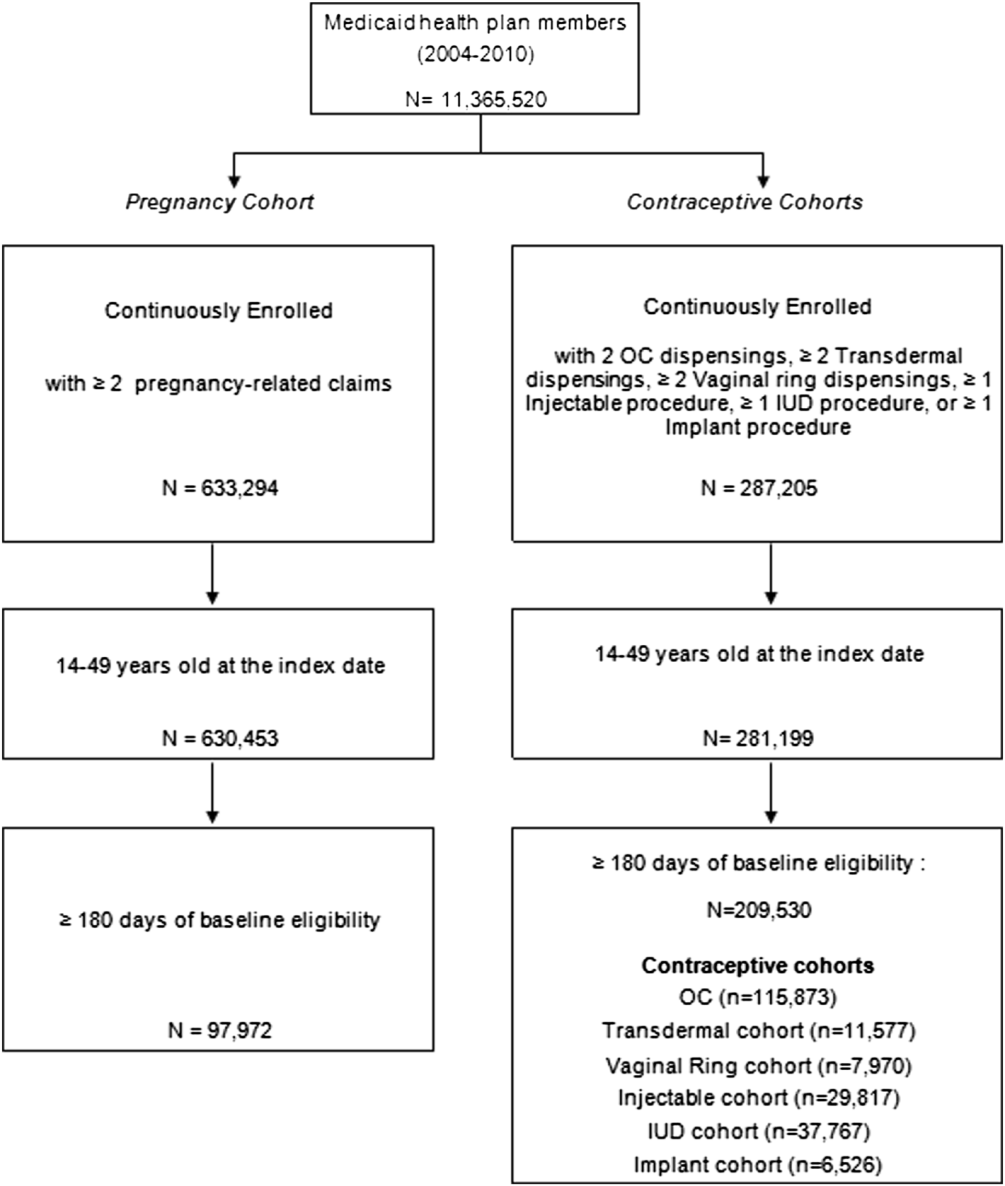
Patients’ disposition flow chart for retrospective cohort analysis.

Figure 1 summarizes the study cohort selection. SARC users (OC: 115,873; transdermal: 11,577; vaginal ring: 7,970; injectable: 29,817) and LARC users (IUD: 37,767; implant; 6,526) were identified. For the pregnancy cohort, a total of 97,972 pregnant women were identified. Table 2 presents the baseline characteristics of the contraceptive and pregnancy cohorts.

**Table 2:** Demographics and clinical characteristics of the study cohorts

	**Index contraceptive**						
**Patient characteristics**	**SARC**				**LARC**		**Pregnancy cohort n = 97,972**
	**OC n = 115,873**	**Transdermal n = 11,577**	**Vaginal ring n = 7,970**	**Injectable n = 29,817**	**IUD n = 37,767**	**Implant n = 6,526**	
**Demographics at index date**							
Age, years, mean (±SD) [Median]	22.7 (7.0) [21]	22.3 (6.4) [21]	24.0 (5.6) [23]	22.6 (7.2) [21]	24.9 (5.6) [24]	21.7 (5.1) [21]	23.3 (6.7) [21]
Age distribution, years, n (%)							
14-16 yrs	22,626( 19.5%)	2,145 (18.5%)	378 (4.7%)	6,037 (20.2%)	491 (1.3%)	871 (13.3%)	10,158 (10.4%)
17-19 yrs	24,784 (21.4%)	2,555 (22.1%)	1,306 (16.4%)	6,332 (21.2%)	4,278 (11.3%)	1,575 (24.1%)	27,917 (28.5%)
20-24 yrs	30,136 (26.0%)	3,275 (28.3%)	3,110 (39.0%)	8,112 (27.2%)	16,480 (43.6%)	2,559 (39.2%)	24,194 (24.7%)
25-29 yrs	19,451 (16.8%)	2,039 (17.6%)	1,998 (25.1%)	4,627 (15.5%)	9,748 (25.8%)	1,047 (16.0%)	17,864 (18.2%)
30-34 yrs	10,162 (8.8%)	926 (8.0%)	765 (9.6%)	2,259 (7.6%)	4,028 (10.7%)	297 (4.6%)	10,205 (10.4%)
35-39 yrs	5,170 (4.5%)	409 (3.5%)	292 (3.7%)	1,342 (4.5%)	1,856 (4.9%)	130 (2.0%)	5,335 (5.4%)
40-44 yrs	2,487 (2.1%)	171 (1.5%)	97 (1.2%)	744 (2.5%)	668 (1.8%)	36 (0.6%)	1,903 (1.9%)
45-49 yrs	1,057 (0.9%)	57 (0.5%)	24 (0.3%)	364 (1.2%)	218 (0.6%)	11 (0.2%)	396 (0.4%)
**Region, n (%)**							
New Jersey	28,532 (24.6%)	3,754 (32.4%)	2,670 (33.5%)	3,976 (13.3%)	3,414 (9.0%)	196 (3.0%)	31,391 (32.0%)
Florida	21,518 (18.6%)	2,142 (18.5%)	1,507 (18.9%)	3,102 (10.4%)	11,067 (29.3%)	590 (9.0%)	16,138 (16.5%)
Missouri	40,641 (35.1%)	3,239 (28.0%)	2,347 (29.4%)	7,381 (24.8%)	12,923 (34.2%)	2,912 (44.6%)	22,716 (23.2%)
Kansas	7,083 (6.1%)	571 (4.9%)	274 (3.4%)	3,022 (10.1%)	2,801 (7.4%)	328 (5.0%)	4,946 (5.0%)
Iowa	18,099 (15.6%)	1,871 (16.2%)	1,172 (14.7%)	12,336 (41.4%)	7,562 (20.0%)	2,500 (38.3%)	22,781 (23.3%)
**Race, n (%)**							
White	72,948 (63.0%)	5,669 (49.0%)	4,186 (52.5%)	13,168 (44.2%)	22,335 (59.1%)	3,534 (54.2%)	46,802 (47.8%)
Black	22,065 (19.0%)	3,723 (32.2%)	2,263 (28.4%)	9,532 (32.0%)	5,914 (15.7%)	1,211 (18.6%)	30,324 (31.0%)
Latin/Hispanic	9,434 (8.1%)	1,077 (9.3%)	735 (9.2%)	1,430 (4.8%)	4,373 (11.6%)	289 (4.4%)	8,704 (8.9%)
Others/Unknown	11,426 (9.9%)	1,108 (9.6%)	786 (9.9%)	5,687 (19.1%)	5,145 (13.6%)	1,492 (22.9%)	12,142 (12.4%)
**Year of index date, n (%)**							
2005	17,725 (15.3%)	5,209 (45.0%)	702 (8.8%)	4,602 (15.4%)	2,724 (7.2%)	14 (0.2%)	14,641 (14.9%)
2006	22,251 (19.2%)	2,201 (19.0%)	1,548 (19.4%)	5,977 (20.0%)	4,738 (12.5%)	32 (0.5%)	18,464 (18.8%)
2007	24,927 (21.5%)	1,529 (13.2%)	1,919 (24.1%)	6,158 (20.7%)	7,790 (20.6%)	696 (10.7%)	20,609 (21.0%)
2008	27,136 (23.4%)	1,393 (12.0%)	1,934 (24.3%)	6,532 (21.9%)	11,813 (31.3%)	2,186 (33.5%)	24,030 (24.5%)
2009	16,850 (14.5%)	890 (7.7%)	1,339 (16.8%)	4,750 (15.9%)	7,208 (19.1%)	2,287 (35.0%)	14,335 (14.6%)
2010	6,984 (6.0%)	355 (3.1%)	528 (6.6%)	1,798 (6.0%)	3,494 (9.3%)	1,311 (20.1%)	5,893 (6.0%)
**Charlson comorbidity index**^**1**^**, n (%)**							
0	103,869 (89.6%)	10,576 (91.4%)	7,222 (90.6%)	26,381 (88.5%)	34,189 (90.5%)	6,031 (92.4%)	89,246 (91.1%)
1	10,013 (8.6%)	832 (7.2%)	656 (8.2%)	2,646 (8.9%)	3,070 (8.1%)	436 (6.7%)	7,054 (7.2%)
2	1,348(1.2%)	109 (0.9%)	60 (0.8%)	474 (1.6%)	363 (1.0%)	36 (0.6%)	972 (1.0%)
3	299 (0.3%)	17 (0.1%)	13 (0.2%)	118 (0.4%)	70 (0.2%)	12 (0.2%)	234 (0.2%)
4	72 (0.1%)	9 (0.1%)	0 (0.0%)	36 (0.1%)	21 (0.1%)	2 (0.0%)	66 (0.1%)
5 or more	272 (0.2%)	34 (0.3%)	19 (0.2%)	162 (0.5%)	54 (0.1%)	9 (0.1%)	400 (0.4%)
**Other comorbidities**^**1**^**, n (%)**							
Cardiovascular diseases	2,737 (2.4%)	215 (1.9%)	179 (2.2%)	823 (2.8%)	1,135 (3.0%)	153 (2.3%)	1,607 (1.6%)
Hypertension	3,125 (2.7%)	202 (1.7%)	199 (2.5%)	974 (3.3%)	1,246 (3.3%)	134(2.1%)	2,182 (2.2%)
Diabetes	2,705 (2.3%)	192 (1.7%)	150 (1.9%)	697 (2.3%)	1,037 (2.7%)	97 (1.5%)	1,703 (1.7%)
Pelvic inflammatory disease	8,851 (7.6%)	945 (8.2%)	843 (10.6%)	2,511 (8.4%)	3,704 (9.8%)	475 (7.3%)	7,350 (7.5%)
Alcohol	697 (0.6%)	49 (0.4%)	42 (0.5%)	270 (0.9%)	161 (0.4%)	30 (0.5%)	753 (0.8%)
Smoking	5,630 (4.9%)	539 (4.7%)	546 (6.9%)	2,035 (6.8%)	3,206(8.5%)	549 (8.4%)	2,945(3.0%)
**Parous status**^**2**^**, n (%)**	50,312 (43.4%)	5,072 (43.8%)	4,541 (57.0%)	13,709 (46.0%)	31,802 (84.2%)	4,204 (64.4%)	2,998 (3.1%)
**Baseline healthcare payments**^**1**^**, dollars, mean (±SD)**							
Inpatient services	$1,615 (4,453)	$1,411 (4,133)	$1,634 (3,344)	$1,554 (4,482)	$2,648 (3,770)	$1,774 (3,295)	$169 (3,021)
Outpatient	$1,574 (4,524)	$1,413 (4,279)	$1,106 (2,009)	$1,856 (4,947)	$1,539 (1,796)	$1,469 (1,930)	$872 (2,461)
Long-term care^3^ services	$78 (2,919)	$32 (1,810)	$0 (6)	$56 (1,881)	$14 (1,142)	$0 (18)	$29 (1,697)
Emergency room services	$37 (161)	$36 (138)	$30 (114)	$49 (190)	$34 (126)	$46 (161)	$40 (227)
Pharmacy	$298 (1,554)	$221 (924)	$181 (919)	$302 (1,409)	$170 (750)	$317 (927)	$204 (1,031)
Total payments	$3,602 (7,598)	$3,113 (6,951)	$2,951 (4,236)	$3,818 (7,826)	$4,405 (4,560)	$3,606 (4,269)	$1,315 (4,914)
**Observation period**^**4**^**, days, mean (±SD)**	317 (91)	324 (88)	316 (90)	325 (90)	265 (133)	305 (101)	337 (63)

Notes: SARC: short-acting reversible contraceptive; LARC: long-acting reversible contraceptive; OC: oral contraceptive; IUD: intrauterine device.

1. Based on a period of 180 days prior to index date.

2. Identified by claims with ICD-9-CM codes for delivery any time prior to the index date. The mother status, available in FL, was also used to identify parous status, as well as the family ID in NJ.

3. Identified with service claims for long-term care, skilled nursing facility, intermediate care facility, and swing beds.

4. From the index date to the earliest date between end of eligibility, end of data availability, or 12 months after the index date.

Mean age was 22.7 years for OC users, 22.3 years for transdermal users, 24.0 years for vaginal ring users, 22.6 years for injectable users, 24.9 years for IUD users, and 21.7 years for implant users, while the mean age for the pregnancy cohort was 23 years. It has been estimated that around 50% of women under 25 years rely on OC for contraception in the US, while the corresponding proportion for women aged 40–44 is only around 10% of women [27]. The mean age of 22.7 years we found for the OC group is thus in a range that is representative of what we would expect for OC users in the US. IUDs and implants have been found to be used mostly by women aged 25–39, married and cohabiting women, women covered by Medicaid, and women with no religious affiliation [28]. Since women covered by Medicaid have been identified as a group likely to use IUDs and implants, it might explain the relatively young age of 24.9 and 21.7 years for IUD and implants users, respectively, that we found in the current study. The mean age for the birth of the first child in the US has been estimated at 25 years old [29], while the mean age of women in the pregnancy cohort in our study was lower at 23.3 years.

In general, IUDs tend to be used by parous women [30]. In the current study we found a qualitatively higher proportion of women who had a parous status at baseline among IUD users (84.2%) compared to implant (64.4%), OC (43.4%), transdermal (43.8%), vaginal ring (57.0%), and injectable methods (46.0%).

The mean follow-ups were 317 days for OC users, 324 days for transdermal users, 316 days for vaginal ring users, 325 days for injectable users, 265 days for IUD users, and 305 days for implant users days, respectively (11 months for SARC users and 9–10 months for LARC users).

### Switch from the index contraceptive

Within 12 months of the index date, the proportion of SARC users switching from index medication to another type of contraceptive was 5.2% for OC users, 18.1% for transdermal users, 10.4% for vaginal ring users, and 9.9% for injectable users. The corresponding proportion of LARC users switching from index medication to another contraceptive was 5.2% for IUD users and 9.1% for implant users.

### Healthcare payments associated with contraceptives and pregnancy

All-cause healthcare payments and payments for contraceptive users are presented in Table 3. Mean all-cause and contraceptive payments PPPM were respectively $365 and $18.3 for OC users, $308 and $19.9 for transdermal users, $215 and $21.6 for vaginal ring users, $410 and $8.8 for injectable users, $194 and $36.8 for IUD users, and $237 and $29.9 for implant users.

**Table 3:** **All-cause and contraceptive healthcare payments per patient per month (PPPM) for contraceptive users**^**1**^

**Variable**	**Healthcare payments per patient per month (PPPM)**					
	**All-cause payments**	**All-cause payments difference relative to OC**		**Contraceptive payments**^**3**^	**Contraceptive payments difference relative to OC**	
		**Unadjusted**	**Adjusted**^**2 **^**(95% CI)**		**Unadjusted**	**Adjusted**^**2 **^**(95% CI)**
Index contraceptive						
OC (N = 115,873)	$365	Reference	Reference	$18.3	Reference	Reference
Transdermal (N = 11,577)	$308	-$57.1	-$4.4 (−17.5;8.6)	$19.9	$1.7	$3.6 (3.1; 4.0)
Vaginal ring (N = 7,970)	$215	-$150	$7.5 (−6.5;23.6)	$21.6	$3.3	$5.1 (4.6; 5.6)
Injectable (N = 29,817)	$410	$45.1	$8.2 (−2.1;18.3)	$8.8	-$9.4	-$9.6 (−9.8; −9.4)
IUD (N = 37,767)	$194	-$171	-$51.8 (−58.1;-45.0)	$36.8	$18.6	$14.9 (14.5; 15.3)
Implant (N = 6,526)	$237	-$128	-$52.5 (−64.2;-41.1)	$29.9	$11.6	$7.4 (6.6; 8.1)

Notes: 1. From the index date until the earliest date between health plan disenrollement, lack of follow-up data, or 12 months after the index date.

2. Adjusted for age, region, race, year of index date, charlson comorbidity index, other comorbidities, parous status, and baseline healthcare payments.

3. Payments for any contraceptives (OC, IUD, transdermal, ring, injectable, and implant).

Despite important variance in unadjusted all-cause healthcare payments among contraceptive users, adjusted all-cause payments PPPM of vaginal ring, transdermal, and injectable users were not significantly different relative to OC users, with payment differences of -$4.4 (−17.5;8.6), $7.5 (−6.5;23.6), and $8.2 (−2.1;18.3), respectively. In contrast, adjusted all-cause healthcare payments PPPM for IUD and implant users were significantly lower compared to OC users, with differences of -$51.8 (−58.1;-45.0) and -$52.5 (−64.2;-41.1), respectively. These lower all-cause payments for LARC users were observed even if they had higher adjusted contraceptive payments compared to OC users, with PPPM differences of $14.9 (14.5;15.3) for IUD users and $7.4 (6.6;8.1) for implant users relative to OC users.

Table 4 reports healthcare payments for the pregnancy cohort. All-cause mean healthcare payments PPPM of the pregnancy cohort were $610, including $391 for claims directly related to pregnancy, which represents 64% of all-cause payments. Almost 90% of the total pregnancy and pregnancy-related payments ($344 of $391) were attributable to pregnancy-related complications.

**Table 4:** **Healthcare payments for the pregnancy cohort (n = 97,972)**^**1**^

**Variable**	**Healthcare payments per patient per month (PPPM)**
Healthcare payments, dollars	
All-cause	$610
Pregnancy-related	$391
Pregnancy-related complication	$344

Notes:

1. From the index date until the earliest date between health plan disenrollment, lack of follow-up data, or 12 months after the index date.

### Actuarial analysis

**Figure 2:**
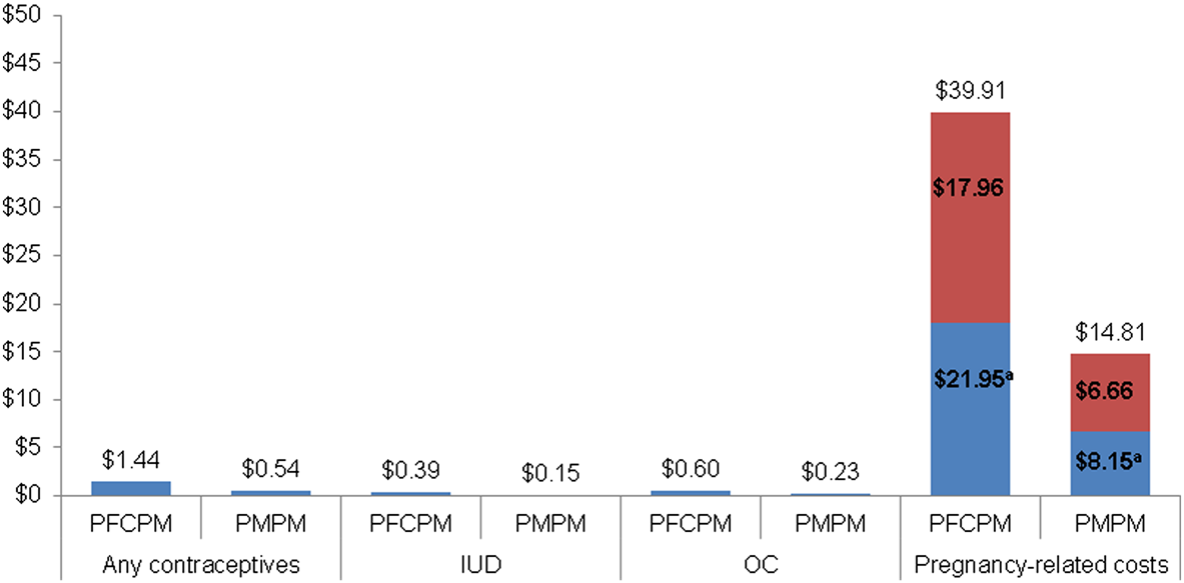
**Payments PFCPM and PMPM for all contraceptives, IUD, OC, and pregnancy in 2008.** Notes: PFCPM: per female member of childbearing age per month; PMPM: per member per month; OC: oral contraceptive; IUD: intrauterine device a. Unintended pregnancies were estimated at 55% based on an article by Finer and colleagues (Unintended Pregnancy Rates at the State Level, Perspectives on Sexual and Reproductive Health 2011; 43:78-87).

A total of 7,031,223 Medicaid members and 2,712,765 women of childbearing age were identified in 2008. Among women aged 14 to 49 years, OC and IUD payments were made for 4.6% (123,955/2,712,765) and 1% (28,399/2,712,765) of women, respectively, while 13.4% (363,968/2,712,765) had payments for pregnancy-related services (data not shown).Payments PFCPM for all contraceptives, IUD, OC, and pregnancy were $1.44, $0.39, $0.60, and $39.91, respectively (Figure 2). Payments PFCPM for all contraceptives, IUD, and OC thus represented a small fraction of the estimated payments for pregnancy at 3.61% ($1.44/$39.91), 0.98% ($0.39/$39.91), and 1.50% ($0.60/$39.91), respectively. Similar proportions were found between contraceptives and pregnancy payments when evaluated PMPM at 3.65% ($0.54/$14.81), 1.01% ($0.15/$14.81), and 1.55% ($0.23/$14.81) for all contraceptives, IUD, and OC, respectively.

Payments for unintended pregnancy were estimated at $21.95 PFCPM and at 37% of that amount ($8.15) when evaluated PMPM. Payments PFCPM for all contraceptives, IUD, and OC also represented a small proportion of the estimated payments for unintended pregnancy at 6.56% ($1.44/$21.95), 1.78% ($0.39/$21.95), and 2.73% ($0.60/$21.95), respectively. The corresponding proportion PMPM for all contraceptives, IUD, and OC where estimated at 6.63% ($0.54/$8.15), 1.84% ($0.15/$8.15), and 2.82% ($0.23/$8.15), respectively.

## Discussion

This large study of Medicaid beneficiaries reported the spending on contraceptive users and pregnancy-related healthcare payments and showed higher all-cause healthcare payments for pregnant women compared to contraceptive users. The study also showed that payments PFCPM and PMPM for contraceptives represented a small fraction of the estimated payments for pregnancy; this finding was expected given that pregnancy and delivery are among the most expensive conditions for insurers. Our study results suggest that coverage of the costs of contraception lower overall expected costs for a health plan. Given that about two-thirds of women covered by Medicaid are of childbearing age [31], the high level of unintended pregnancies [5], and the fact that Medicaid covers around two thirds of these pregnancies [3,4], access to adequate contraceptive coverage fills a key public health care need.

Although the purpose of this research was not to study the impact of the ACA, these findings are particularly relevant given that the Medicaid expansions under the ACA will result in much larger numbers of women being eligible for coverage by Medicaid, at least in those states that choose to expand their Medicaid programs [2]. These results are also of importance for states that have not yet decided to enter the ACA as they showed potential cost-offsets that could result in more women being eligible for coverage by Medicaid (including contraceptive coverage), especially given the high number of unintended pregnancies among Medicaid beneficiaries [5].

Previous studies have reported savings for contraceptive coverage use relative to pregnancy and maternity care [14-16]. Family PACT, California’s publicly-funded family planning program, estimated savings of more than $7.00 for every $1.00 spent on services and supplies for implant and intrauterine contraceptives, while $1.00 spent for injectable contraceptives translated to savings of $5.60; $4.07 for oral contraceptives; $2.99 for the patch; and $2.55 for the vaginal ring [14]. A study by Frost and colleagues estimated public expenditure savings for family planning care of $4.02 for every dollar spent [15]. A study by Trussell and colleagues concluded that the least expensive and most cost-effective of the examined methods were the IUD and vasectomy, and that any mode of contraception is less expensive than no method [16]. In the current study, we found similar trends: all-cause healthcare payments PPPM of pregnant women were higher compared to those of women using contraceptives after up to one year of follow-up (11 months for SARC users and 9–10 months for LARC users). Furthermore, LARC users were associated with lower adjusted all-cause payments compared to OC users despite higher contraceptive payments associated with the initial dispensing and insertion of LARC. Of note, PPPM contraceptive payments for LARC users are related to the chosen follow-up period since these payments all occurred upfront. The PPPM payments for LARC contraceptive payments reported in the current study are thus substantially higher than if they were calculated for a longer follow-up (e.g., five years).

A previous actuarial analysis similar to the current study was conducted on a commercially insured population [25]. Using the MedStat Marketscan Database for the year 2008, the authors found that costs PMPM for IUDs and OCs represented 1.8% ($0.26/$14.64) and 11.7% ($1.72/$14.64) of the cost of pregnancy, respectively. In the current study, the payments PMPM of IUDs and OCs represented an even lower proportion of the payments of pregnancy at 1.0% ($0.15/$14.81), and 1.5% ($0.23/$14.81), respectively. The differences between the two studies could be partly explained by the different study populations; the study by Fitch and colleagues was based on a commercially insured population, while our study was based on a Medicaid population. Nevertheless, both studies agree in the general finding that contraceptive coverage represents a small proportion of pregnancy costs.

Moreover, the current findings can be used to highlight potential cost savings of contraception, including LARC methods, relative to costs incurred by unintended pregnancies. As an example, contraceptive payments among IUD users were estimated at $36.80 PPPM compared to $21.95 for unintended pregnancy PFCPM as estimated from the actuarial analysis. Given that IUDs are expected to last several years, these results suggest that cost savings associated with the use of IUDs to prevent unintended pregnancy may be achieved after only two years, as payments for IUDs occur up front and were calculated based on up to 1 year of observation in our study (i.e., $36.80/2 years ≈ $18.40 PPPM). From a policy perspective, because we cannot distinguish between intended and unintended pregnancies, if we assume that all women of childbearing age had used an IUD and had no contraceptive failure, this would represent cost savings of $3.55 PFCPM ($21.95-$18.40) in 2008, or $1.37 PMPM on the whole Medicaid population. Similar calculations for implant users and SARC users also result in potential cost savings. Of course, in real life, not all women would or should use an IUD or implant; many women would prefer to choose a different type of contraception; some women would not use any contraception at all, and some women would want to become pregnant. Nevertheless, these potential savings from preventing unintended pregnancies further support a need to provide women with improved and affordable access to and choice between all available contraceptive methods, including LARC methods.

A study of the 2002 National Survey of Family Growth revealed that women aged 18 to 24 at risk of unintended pregnancy were three times more likely to use a prescription contraceptive when insured with Medicaid or with private insurance than when uninsured [32]. Other studies support the finding that fewer uninsured women at risk for unintended pregnancies use prescribed contraceptives compared to publicly or privately insured women [33,34]. Although Medicaid federal guidelines require all states to cover family planning services, they do not specify which services must be provided. A nationwide state survey conducted from Spring 2007 through Winter 2008 found that 32 states covered all types of prescription contraception as a family planning service under Medicaid, 12 states sometimes considered all of them as family planning (4 did not always consider OC under all circumstances), and 7 states did not respond to the survey [35]. Among the states included in the current study, Florida, Iowa, and Kansas included all forms of contraceptives under the definition of family planning services, Missouri excluded IUD removal, whereas New Jersey did not respond to the survey. Thus, inconsistent coverage of contraception under family planning services has likely resulted in decreased access and/or utilization and thus more unintended pregnancies.

This study has several limitations. First, claims data may have inaccuracies in the recorded information (e.g., diagnoses, payments). Second, there may be variations in the coverage of contraceptive care among the five states included in our Medicaid database; any such differences were not taken into account in the analysis. Third, the study evaluated only the direct medical payments of pregnancy. Information to determine the indirect costs of pregnancy, such as work productivity loss, was not available, and we did not include the costs of newborn care. Fourth, the observational design was susceptible to various biases. For example, contraceptives purchased over-the-counter were not in the database, which may have resulted in an underestimation of contraceptive payments. Finally, because exposure to contraceptives was not randomly assigned across patients, there is the possibility of confounding by indication. Despite these limitations, the current research has several advantages, including the importance of relying on real-world data and a relatively large sample size.

## Conclusion

This large retrospective study of Medicaid female members aged 14 to 49 years old showed that, over a follow-up period of 12 months, healthcare payments for pregnancy are considerably higher than payments for SARC and LARC users. Healthcare payments for contraceptive methods represented a small proportion of unintended pregnancy payments when considered from the overall Medicaid population perspective over a full calendar year.

## Competing interests

Three of the authors (Laliberté F, Lefebvre P, and Duh MS) are employees of Analysis Group, Inc., a consulting company that has received research grants from Bayer HealthCare Pharmaceuticals Inc. Three of the authors (Law A, Pocoski J, and Lynen R) are employees of Bayer HealthCare Pharmaceuticals Inc. Dr. Darney has received research grants from Bayer HealthCare Pharmaceuticals Inc. Bayer is the manufacturer of the Mirena® and Skyla™.

Parts of this manuscript were presented at Women’s Health 2013: The 21st Annual Congress, Washington, DC, March 22–24, 2013 and at the First Global Conference on Contraception, Reproductive and Sexual Health, Copenhagen, May 22–25, 2013.

## Authors’ contributions

All authors participated to the study concept and design and data interpretation. FL performed the data collection and statistical analyses. Writing of the manuscript was shared by FL, PL, and MSD. Revision of the manuscript was shared by PD, AL, JP, and RL. All authors read and approved the final manuscript.
